# New Insights into the Link between SARS-CoV-2 Infection and Renal Cancer

**DOI:** 10.3390/life14010052

**Published:** 2023-12-28

**Authors:** Vittoria Rago, Sabrina Bossio, Danilo Lofaro, Anna Perri, Silvia Di Agostino

**Affiliations:** 1 Department of Pharmacy, Health and Nutritional Sciences, University of Calabria, 87036 Rende, Italy; vittoria.rago@unical.it; 2 Department of Experimental and Clinical Medicine, Magna Græcia University of Catanzaro, 88100 Catanzaro, Italy; sabrina.bossio@unicz.it; 3 de-Health Lab, Department of Mechanical, Energy, Management Engineering, University of Calabria, 87036 Rende, Italy; danilo.lofaro@unical.it; 4 Department of Health Sciences, Magna Græcia University of Catanzaro, 88100 Catanzaro, Italy; sdiagostino@unicz.it

**Keywords:** SARS-CoV-2 infection, COVID-19, renal cancer cell (RCC), ACE2, TMPRSS2, NRP1

## Abstract

Cancer has been described as a risk factor for greater susceptibility to SARS-CoV-2 infection and severe COVID-19, mainly for patients with metastatic disease. Conversely, to that reported for most solid and hematological malignancies, the few available clinical studies reported that the infection did not increase the risk of death in renal cancer patients. The expression on proximal tubular renal cells of the key players in cellular viral uptake, ACE2, TMPRSS2, and NRP1, seems to be the mechanism for the direct kidney injury seen in patients with COVID-19. Interestingly, data from The Cancer Genome Atlas and experimental analyses on various renal cancer cell lines demonstrated that the above-reported receptors/cofactors are maintained by renal cancer cells. However, whether SARS-CoV-2 infection directly kills renal cancer cells or generates enhanced immunogenicity is a question worth investigating. In addition, some researchers have further addressed the topic by studying the expression and prognostic significance of gene signatures related to SARS-CoV-2 infection in renal cancer patients. The emerging data highlights the importance of better understanding the existence of a link between renal cancer and COVID-19 since it could lead to the identification of new prognostic factors and the development of new therapeutic targets in the management of renal cancer patients.

## 1. Introduction

SARS-CoV-2, the virus responsible for the coronavirus disease 2019 (COVID-19), has resulted in unprecedented morbidity and mortality in the world. Epidemiological and clinical data reported that symptoms can last up to four weeks after infection, or they can present four to 12 weeks after the acute phase of COVID-19, or the disease persists for a long time leading to long COVID (LC) [1]. LC is also associated with pre-existing pathologies, such as respiratory and cardiovascular diseases, advanced age, high body mass index, or previous history of comorbidities such as chronic kidney injury, and cancer [2]. In particular, growing clinical data have reported that renal function impairment represents a relevant complication requiring long follow-up by nephrologists [3,4,5,6].

The risk of developing a serious disease in cancer patients with COVID-19 is about 4 times higher compared to those without malignancy [7], because of compromised immune systems caused by tumor depletion, malnutrition, and anticancer treatments. In addition, SARS-CoV-2 can activate the metabolism of tumor cells, such as increasing glycolysis which in turn can facilitate virus replication [8,9] and alter the metabolic pathways of cancer cells, accelerating tumor progression [10]. In vitro studies performed by infecting the human colon cell line (Caco-2) with SARS-CoV-2 have demonstrated that the infection alters central cellular pathways, such as translation, splicing, carbon, nucleic and acid metabolism [11], blocking viral replication [12]. In addition, some authors have demonstrated that 24 h after SARS-CoV-2 infection, the proteomics of Caco-2 cells undergoes an extensive alteration, leading to a decrease in metabolic proteins rich in cholesterol and an increase in proteins that modify carbohydrate metabolism [11]. In this way, SARS-CoV-2 infection could reprogram metabolism in tumor cells to facilitate viral replication and influence tumor progression [13,14]. Epidemiological studies showed that COVID-19 infection does not affect the mortality rate among patients with early-stage cancer [15], whereas, patients with advanced cancer are not only more likely to be infected by SARS-CoV-2 but also have a worse prognosis, especially if male [16]. Most patients hospitalized for COVID-19, have acute kidney injury (AKI) and although the pathophysiology of AKI in these patients is not fully understood, the studies suggest that AKI, as well as chronic kidney disease, are associated with poor survival [5,17] and with an increased incidence of renal cancer [18]. The frequent renal involvement with organ damage in patients with COVID-19 is undoubtedly multifactorial, but the expression on renal cells of the key receptors promoting SARS-CoV-2 entry, such as ACE2, TMPRSS2, and NRP1 can make the kidney particularly susceptible to SARS-CoV-2 infection [19,20,21]. It is well known that proximal tubular renal cells are cells of origin of the major types of renal cell carcinoma (RCC), clear cell renal cell carcinoma (ccRCC 75–80%), and papillary RCC (pRCC; 10–15%) [22]. The etiology of these neoplasms and the underlying biological mechanisms responsible for the onset and progression of the disease are still to be understood [23], although it is now known that several altered signaling pathways contribute to RCC pathogenesis and drug resistance [24,25,26,27]. Interestingly, emerging evidence suggests that the retained expression of angiotensin-converting enzyme 2 (ACE2), transmembrane serine protease 2 (TMPRSS2), and neurolipin 1 (NRP1) on the renal cancer cell could increase tumor immunogenicity and promote a cytolytic effect [28,29]. The available data concerning the latter topic is still speculative and preliminary, but some authors suggest that it could open a new scenario in the development of selective drugs targeting NRP1, TMPRSS2, or ACE2 in the treatment of RCC.

This narrative review aims to describe the main studies exploring the cellular mechanisms conferring to renal cancer patients a potentially greater susceptibility to SARS-CoV-2 infection. In addition, the emerging findings investigating the clinical implications of ACE2, TMPRSS2, and NRP1 expression on renal cancer cells for RCC treatment, and the prognostic significance of gene signatures related to SARS-CoV-2 infection in renal cancer cell patients will be reviewed.

## 2. Materials and Methods

The current state of knowledge reported in this narrative review has been conducted using PubMed and Google Scholar. We selected original articles (in vitro and human studies), and reviews in the English language published between January 2020 and October 2023, using the search terms “COVID-19 severity AND cancer”, “COVID-19 AND Renal cancer”, SARS-CoV-2 infection AND renal cancer”, “SARS-CoV-2 entry AND renal cells”, ACE2 OR TMPRSS2 OR NRP1 AND renal cells”, SARS-CoV-2 AND renal cancer cell, “SARS-CoV-2-related genes AND renal cancer. The article selection was based on the article title and abstract; then the full text was critically evaluated and included in the review.

## 3. Expression of SARS-CoV-2 Entry Factors in Normal and Cancer Renal Cells

The expression of SARS-CoV-2 entry factors in normal and cancerous kidney cells has been a subject of research interest, especially in the context of understanding the potential for COVID-19 infection in individuals with kidney cancer or kidney-related diseases. SARS-CoV-2, as previously described enters human cells mainly through the interaction of its spike protein with the key cellular receptors, ACE2, TMPRSS2, and NRP1 [30,31,32]. The presence and expression levels of these receptors, in combination with many other co-factors on the surface of kidney cells, can influence the susceptibility of these cells to SARS-CoV-2 infection.

The co-expression of ACE2, TMPRSS2, and NRP1 in the proximal tubules of normal kidney tissue should assist the infection of the virus and it could represent a mechanism for the direct kidney damage reported in patients affected by COVID-19 [33]. The expression of ACE2 and TMPRSS2 in kidney cells has raised questions about the susceptibility of individuals with kidney-related diseases, including kidney cancer, to SARS-CoV-2 infection. Patients with pre-existing kidney disease may have altered expression of these factors due to their underlying disease, which could potentially influence the course and outcome of COVID-19 if they become infected.

### 3.1. Angiotensin-Converting Enzyme 2 (ACE2)

ACE2 is the primary receptor for SARS-CoV-2 [34]. It is known to be expressed in various tissues throughout the body, including the lungs, blood vessels, liver, and gastrointestinal tract, and at high levels in the kidneys and heart [35]. At the physiological level, ACE2 is involved in regulating blood pressure and plays a role in the renin-angiotensin-aldosterone system (RAS) where the overactivation of the RAS pathways results in hypertension, kidney damage, and cardiovascular disease, in addition to being one of the pivotal signaling in SARS-CoV-2–induced nephrotic syndrome [36]. ACE2 is a zinc metalloenzyme and carboxypeptidase expressed on the surface of endothelial cells and other cell types. Although its primary substrate is angiotensin type II, it can hydrolyze numerous other physiological substrates [36]. The enzymatic activity of ACE2 cleaves the C-terminal end of angiotensin II, producing the 7 amino acid peptide Ang(1–7). The binding of Ang(1–7) to the Mas receptor (MasR) antagonizes numerous effects of Ang II in a negative feedback loop mechanism [37].

At the cellular level, angiotensin II induces various signaling pathways, including serine/threonine kinase, ERK, JNK/MAPK, and PKC [38]. The activation of these pathways through G protein-coupled receptors induces downstream cytokines IL-6 (interleukin 6) and TNF-α (tumor necrosis factor-alpha) [39,40], important mediators of inflammatory processes and the antibody immune response. Of interest to COVID-19 disease, angiotensin II can increase inflammation and cell death in the lung alveoli resulting in a dramatic decrease in the amount of oxygen in the body [37]. These harmful effects of angiotensin II are reduced by the activity of ACE2, which works as a key protective enzyme by reducing inflammation, oxidative stress, and fibrotic processes.

In the kidneys, ACE2 is primarily expressed in renal tubular epithelial cells, particularly in the proximal tubules. Research has shown that ACE2 expression is upregulated in certain kidney diseases, such as diabetic nephropathy and chronic kidney disease. This has led to investigations to decrease ACE2 levels and/or use MasR agonists/antagonists to modulate the activity of the ACE2-Ang(1–7)-MasR axis to improve renal and cardiovascular disease [41]. However, relatively little effort has been expended in recent years to characterize the function of ACE2 in the human kidney, despite the significance of ACE2 in the pathogenesis of chronic kidney disease. With the research on the mechanisms of SARS-CoV-2 virus infection, some groups have begun more in-depth studies of the function of ACE2 in the kidney under physiological conditions [42,43].

ACE2 has been reported to be a tumor suppressor gene in diverse cancers, however, in breast invasive and thyroid carcinoma, in prostate cancer, liver hepatocellular carcinoma, kidney chromophobe, and stomach adenocarcinoma it was considered an oncogene [15]. However, studies examining ACE2 expression in renal cancer are very few, many showed in silico analysis and overall have reported mixed results.

ccRCC is the most common kidney tumor in adults (about 75% of incidence) and originates from the renal tubule [44]. In silico analysis reported that ACE2 was at low expression levels in ccRCC, where the high expression was associated with a favorable overall and disease-free survival [45]. Immune cell infiltration into the tumor is instrumental in the prognosis of renal cancer [46]. The study by Niu and colleagues highlighted that ACE2 mRNA expression showed a positive link to the abundance of B cells, CD8+ T cells, macrophages, neutrophils, and dendritic cells, while the copy number alteration of ACE2 could inhibit the number of infiltrating cells [45]. The inhibitory effect of ACE2 expression on human tumor proliferation in ccRCC was further confirmed by in vitro and in preclinical animal models showing that lower ACE2 expression was also associated with advanced tumor stage (T3/T4), higher histological grade (grade 3/4), pathological stage (III/IV) and metastasis (M1) [47,48].

However, recent research studies reported high ACE2 expression levels, in particular in papillary (94%) and clear cell (86%) renal carcinoma [28,48,49,50]. The variability in ACE2 expression in renal cancer cells has raised questions about whether there could be differences in susceptibility to SARS-CoV-2 infection among individuals with RCC. It’s important to note that ACE2 expression is just one factor in determining susceptibility to infection, and other factors, such as host immune responses, also could play a role.

It has been reported that the *Ace2* gene resides on chromosome 16 and in the kidney it is tightly controlled by an upstream genetic locus on the same chromosome consisting of six genes whose expression is highly correlated with that of ACE2 [48]. The expression of ACE2 and the co-variants are involved in the RAS signaling which has a renal protective function, in particular, ACE2 and PDGFC are co-expressed and play an important role in the renal physiology and morphology [48]. Interestingly the authors mimed the mechanism of SARS-CoV-2/ACE2 binding and the invasion mechanism of host cells by using the CAKI-1 renal cancer cells. SARS-CoV-2 epitope S (spike glycoprotein) was reported to induce the repression of ACE2, PDGFC, and RAS pathway [48]. These interesting findings showed that knowledge of the ACE2 gene regulatory network is important in revealing potential genetic drivers in ACE2-dependent renal function, the results of which are useful for the diagnosis and treatment of renal cancer patients with COVID-19 complications.

A very recent study demonstrated the involvement of communication from the extracellular matrix (ECM) to proximal tubular epithelial cells on ACE2 levels. Integrin α2β1 was shown to be a player in mediating between SARS-CoV-2 entry and ACE2 expression [51]. Normal and kidney tumor cells, which express high levels of ACE2, treated with an α2β1 integrin antagonist showed decreased ACE2 expression and inhibition of viral entry. This study demonstrates that integrin β1 positively regulates the expression of ACE2, necessary for the entry of SARS-CoV-2 into kidney cells [51].

Recently, it was reported that the SARS-CoV-2 virus replicated in renal cell carcinoma cells causing distinct virus cytopathogenic effects [28]. Furthermore, the authors studied one case of clear cell carcinoma in which the patient contracted the SARS-CoV-2 virus 12 months before nephrectomy. Histologically, the cells showed a peculiar fusion into syncytia, discoid in shape with signs of detachment from the basal lamina. Approximately 80% of the tumor was necrotic [28]. Formation of CCRCC cell syncytia was in vitro confirmed in clear cell renal cell carcinoma cells infected by the Delta variant [28]. Similarly, three patients with metastatic colorectal cancer (mCRC) experienced a reduction in disease burden during the COVID-19 coronavirus disease [52]. These data would suggest a potential causal connection in which viral infection of tumor cells can cause lysis of the cells. Accordingly, not only this oncolytic cellular effect is important but also the possibility of an enhanced immune response elicited by tumor cells subjected to viral infection.

Interestingly, in some cases, SARS-CoV-2 infection can reactivate the immune system through cytokine storm-dependent inflammation caused when the virus actively replicates. Such immune reconditioning induces the reactivation of previously exhausted T lymphocytes to reprogram and target tumor cells for destruction (i.e., reduction/remission of acute myeloid/lymphoblastic leukemias) [53]. These cases suggest new and controversial therapeutic approaches where genetically modified SARS-CoV-2 virus, in which virulence is significantly reduced, could be used to infect a cancer patient as a form of treatment [54].

### 3.2. Transmembrane Serine Protease 2 (TMPRSS2)

TMPRSS2 is a cellular protease that primes the spike protein of SARS-CoV-2, enabling the virus to enter host cells more efficiently. TMPRSS2 is also expressed in the kidneys, primarily in the proximal tubules. Its expression in renal cells, like ACE2, can influence the ability of SARS-CoV-2 to infect these cells.

The expression of TMPRSS2 in cancerous kidney cell lines, such as those found in renal carcinoma tissues can vary. There is no in-depth literature on this topic however some studies have shown that the expression of TMPRSS2 can be altered in various types of cancer. A recent in silico study reported that the expression of TMPRSS2 was low in different cancers, such as colon adenocarcinoma, head and neck squamous cell carcinoma, breast invasive carcinoma, kidney renal papillary cell carcinoma, ccRCC, rectum adenocarcinoma, lung cancer, liver hepatocellular carcinoma, skin cutaneous melanoma, stomach adenocarcinoma and others [55]. On the contrary, elevated expression of TMPRSS2 was reported in bladder urothelial carcinoma, cervical squamous cell carcinoma and endocervical adenocarcinoma, cholangiocarcinoma, glioblastoma multiforme, kidney chromophobe, uterine corpus endometrial carcinoma, and pancreatic and prostate adenocarcinoma [55].

At the experimental level, Choong and colleagues analyzed the expression of ACE2, TMPRSS2, and NRP1 proteins in RCC by immunohistochemistry of tissue microarray (TMA) by using 263 cases of CCRCC, 139 of pRCC, 18 of CHRCC, and human kidney tissue as controls [28]. They reported that CCRCC tissues were positive at 76%, 81%, and 85% for ACE2, TMPRSS2, and NRP1 respectively. pRCC showed 93%, 56%, and 66% positivity for ACE2, TMPRSS2, and NRP1. CHRCC was negative for ACE2 and NRP1 but showed a weak TMPRSS2 positivity in 50% of the tissues [28].

Mechanisms of TMPRSS2 downregulation in cancer are not yet known. In Head and neck squamous cell carcinoma (HNSCC), an aberrant upregulation of a group of specific microRNAs has been highlighted that could target TMPRSS2 at a post-transcriptional level reducing its levels in the tumor [56]. Furthermore, the methylation status of the TMPRSS2 promoter does not change between tumors and normal tissues. Interestingly, this anti-correlated expression was also evidenced in an HNSCC patient positive for SARS-CoV-2 infection [56]. It would also be interesting to evaluate this possibility of mechanism in renal cancer where the general expression of TMPRSS2 does not seem to be so high.

The fact that TMPRSS2 expression is low does not lead to an automatic decrease in the possibility of infection. Preclinical studies on prostate cancer have demonstrated that while the pharmacological inhibition of androgen receptor decreases the expression of TMPRSS2, in parallel it induces the expression of ACE2, increasing the risk of SARS-CoV-2 infection [10]. This reasoning can be translated throughout the complex network of regulations within a tumor cell.

The expression of ACE2 and TMPRSS2 in kidney cells has raised questions about the susceptibility of individuals with kidney-related diseases, including kidney cancer, to SARS-CoV-2 infection. Recent studies revealed that other SARS-CoV-2 factors such as ANPEP (alanyl aminopeptidase, membrane), ENPEP (glutamyl aminopeptidase), and DPP4 (dipeptidyl peptidase 4) are expressed at high levels in renal cancer, especially in ccRCC. Furthermore, the presence of these SARS-CoV-2 receptors is strongly associated with immune infiltrates and immune response in ccRCC [57,58]. Hundreds more proteins that interact with SARS-CoV-2 have been identified in the last three years, thickening the net to clarify their expression, patient prognosis, and immunity [59].

### 3.3. SARS-CoV-2 Infection and Renal Cancer: Oncolytic Properties and Improvement of Antitumor Immunity

Oncolytic viruses are a group of viruses that can lead cancer cells toward death, so they are employed for anti-cancer immunotherapy, including therapy for renal cell carcinoma [60,61,62,63]. Very recently Fang and colleagues reported that combined treatment of carbonic anhydrase 9 (CA9)-targeted CAR-T cells with an oncolytic adenovirus carrying the chemokine (CC motif) ligand 5 (CCL5), cytokine interleukin-12 (IL12) induced moderate inhibition of xenografted tumor in nude mice and increased infiltration of CD45+CD3+ T cells and prolongation of mouse survival in immunocompetent mice [64].

While the oncolytic activity of various viruses has been known and has been exploited for years for the therapy of renal cancer [60,61,62,65], practically to date the papers published by searching with the words key “oncolytic AND SARS-CoV-2 AND renal cancer” are two, one of which is unrelated to the topic [28,66].

The exciting question that many research groups are eager to answer is whether SARS-CoV-2 directly affects tumor cells or generates a greater state of immunogenicity. For example, recombinant poliovirus injected into human breast cancer, melanom, and prostate cancer cell models can increase immune activity in the tumor microenvironmental [67]. As previously commented, Choong and colleagues suggested two scenarios: one of an oncolytic type mediated by the virus on ccRCC cells exposed to the SARS-CoV-2 Delta variant and in those of the tumor of the patient with COVID-19 infection followed by nephrectomy; a second where viral infection of tumor cells induces antigen expression and a greater immune response [28]. A recent review describes very well the pathways and molecules involved in these two aspects [29].

Although the results of the expression of SARS-CoV-2 receptors and its interactors in ccRCC and pRCC are clear, the studies conducted so far not only on kidney tumor models but also on other cancers are too few and above all, they make use of in silico analyses which should then be supported by robust validation experiments. Therefore, drawing conclusions regarding potential treatments for RCC by exploiting this preliminary knowledge on the effects of SARS-CoV-2 viral infection is premature. It is necessary to expand the case studies of patients affected by COVID-19 and RCC and increase genome-wide expression analyses which are still very limited.

## 4. Mortality Risk and Severity of COVID-19 in Advanced Renal Cancer Patients

Cancer has been included among the major risk factors for death in patients infected with COVID-19, so during the pandemic, several cancer societies developed and regularly updated specific guidelines for cancer care [68]. However, in the face of this crucial action, it is well known that screening and diagnostic programs, also for cancer care, were severely affected during the pandemic, with consequent higher prevalence of more advanced-stage presentation [69,70,71]. Different factors are responsible for the increased risk of death in patients with cancer and COVID-19, especially in individuals in an advanced stage. Among these influencing factors, undoubtedly, the dysfunction of innate and humoral immunity, depending on therapies with immunosuppressive effects, and the presence of comorbidities, play a key role. The observational prospective study ESMO-CoCARE, including data from 1626 patients with solid/hematological malignancies collected since June 2020, validated previously published observations on variables associated with COVID-19 outcomes in patients with cancer. Interestingly, the authors found that Asian ethnicity and higher BMI are associated with better COVID-19-related outcomes, suggesting that the “obesity paradox”, host genetics, and human leukocyte antigen profiles may influence COVID-19 outcomes [66,72,73,74]. Compared to other cancers, a very less robust body of evidence is currently available regarding the mortality rate of ccRCC patients infected with COVID-19, probably because of the low incidence of this tumor, but the few available retrospective studies did not register any increase in the severity or mortality of COVID-19 infection within this cohort of patients [75].

A widely debated topic in literature is the association between anti-tumor therapies and severity and mortality in cancer patients with COVID-19. Although the immunologic implications and the high proinflammatory status of advanced cancer worsen the severity and mortality of COVID-19 infection in cancer patients, it is undeniable that systemic anticancer therapy increases the level of complexity, further worsening the outcome. Divergent and contradictory results emerge from these studies, probably because of heterogeneity related to the class of therapies, the time variability between treatment and COVID-19 diagnosis, and the type of cancer. Among anticancer treatments, immune checkpoint inhibitors (ICIs) have received particular attention from oncologists during the pandemic, since some pieces of evidence suggest that pulmonary toxicity and the restoration of immunocompetence both ICIs-induced favor the development of cytokine release syndrome and contribute to the severity of COVID-19 [76,77]. On the other hand, recent studies did not observe an increased contraction or mortality of COVID-19 infection among cancer patients receiving ICIs [66,78]. However, it should be considered that the implications of immunotherapy in COVID-19-infected cancer patients could depend on the type of tumors, solid or hematological, because the immune dysfunction, the T-cell response after infection, and the tumor microenvironment are different each from other.

Immunogenicity is a peculiar characteristic of renal cell carcinoma since this neoplasm is often diffusely infiltrated by CD8^+^ T lymphocytes, macrophages, neutrophils, and dendritic cells [79]. Therefore, in recent years, the discovery of ICIs, in monotherapy or combination with other drugs, has revolutionized the treatment of advanced renal cell carcinoma. ICIs work by blocking lymphocyte receptors (such as PD-1 or CTLA-4), namely the ligand on the tumor cell (PDL-1), thus reactivating the physiological anti-tumor response [80,81]. However, just as it occurs for drugs with anti-angiogenic activity, many patients develop resistance to ICIs because the tumor cells can activate alternative inhibitory pathways or create an ‘immune cold’ microenvironment [82,83]. Few clinical studies investigated the impact of COVID-19 in advanced renal cell cancer patients in systemic therapy. In agreement with previous reports [84,85], the recent retrospective case-control study conducted by García-Donas J et al. reported that patients who developed COVID-19 and were treated with antiangiogenics or immunotherapy, required more treatment interruptions and hospitalizations than those non-infected, but no significant impact on cancer outcome was observed [86].

Overall, the data emerging from clinical studies and metanalysis show that the mortality rate associated with COVID-19 for patients with cancer varies from 13% to 33.6%, depending on many factors such as population heterogeneity and a selection bias towards the most severe cases in some studies [66]. Regarding renal cancer, the data, albeit limited and derived from very small study cohorts, show that in these patients, COVID-19 infection does not increase the risk of death. In addition, the recent literature suggests that targeted therapy or immune checkpoint inhibitors have not been associated with worse outcomes from COVID-19, mainly in renal cell cancer patients [66].

For further consideration, it should be kept in mind that now we are facing another phase of the pandemic with a significant proportion of patients with cancer vaccinated against COVID-19, therefore, the future scenario could change in terms of outcomes in these patients.

## 5. SARS-CoV-2–Related Genes as New Potential Prognostic Factors and Therapeutic Targets for Renal Cancer Cell Patients

A growing literature pointed out emerging topics regarding the role of SARS-CoV-2 infection in the progression of RCC and the research of predictive factors of cancer progression and new therapeutic strategies to counteract the complications of COVID-19-related and mortality risk. In ccRCC, using the TCGA dataset, Huang and colleagues identified 31 SARS-CoV-2-related genes differentially expressed between ccRCC and normal kidney tissues [87]. The interesting part of this study is that the authors selected 5 genes that were able to stratify low- and high-risk ccRCC patients with poor survival. Gene set enrichment analysis (GSEA) showed that some inflammatory/immune pathways were significantly enriched in the high-risk group, where patients belonging to the high-risk group had higher stromal and immune cell scores, thus purity of the lower tumor. Furthermore, these high-risk patients had high numbers of M0 macrophages, regulatory T cells, and T follicular helper cells and increased expression of the immune checkpoints CTLA-4, LAG-3, TIGIT, and PDCD1 compared to low-risk patients. In conclusion, this prognostic signature based on genes related to SARS-CoV-2 as reliable prognostic predictors for ccRCC patients is very promising and indicates a precise road of future research that is inseparable from massive sequencing studies [87]. Interestingly, a recent study identified the most common biological pathway correlated to SARS-CoV-2 and ccRCC. The study was conducted by using two different datasets, GSE53757 for ccRCC and GSE164805 for SARS-CoV-2 infected patients; among the COVID-19 patients, the authors found 67 genes upregulated, and 176 genes downregulated, whereas, in the ccRCC dataset they identified 106 genes upregulated and 77 genes downregulated. Furthermore, a gene ontology analysis was conducted to elucidate the important role of DEGs between COVID-19 and ccRCC in biological processes such as mitochondrial matrix, glucose metabolism, and chemokines receptor signaling. Glucose metabolism is important in the regulation of the growth and progression of most types of cancer, but also the glucose levels in the cells are associated with the severity of the SARS-CoV-2 infection. It has been reported that a dysregulation of the glucose metabolism restricts the virus from replication and prevents the activation of cytokines responses [14]. When the pathogens invade the body, the release of cytotoxic factors induces the production of many chemokines [88]. It was observed that COVID-19 patients produced and released higher levels of chemokines due to dysregulated glucose metabolism, contributing to exacerbating the disease and increasing complications and mortality risk [89]. The DEGs obtained by the Kyoto Encyclopedia of Genes and Genomes (KEGG) analysis of the two different datasets showed that DEGs were involved in several processes such as Hypoxia-inducible factor 1-α (HIF-1α) signal transduction and glycolysis. HIF-1α is an important factor for inflammation and metabolic pathways and its upregulation in ccRCC implies a poor prognosis [88]. In addition, HIF-1α induces the replication of the virus and causes inflammation among COVID-19 patients [90]. Therefore, it emerges that HIF-1α signaling, dysregulated glucose metabolism, and chemokine signaling pathway play an important role among cancer patients with COVID-19 infections. Thus, the identification of essential genes for COVID-19-associated metabolic pathways may act as a promising therapeutic target to prevent complications of COVID-19-related among kidney cancer patients [91]. Another study used a bioinformatic approach to analyze the gene and protein expression data of coronavirus receptors (DPP4, ANPEP, ENPEP, TMPRSS2) in human normal and cancer tissues of different organs including kidneys. RNA-seq data obtained between renal tumor and normal tissues indicate that there were increased levels of ACE2, DPP4, ANPEP, and ENPEP receptors. Therefore, it turned out that TMPRSS2 may not be the co-receptor for COVID-19 infection in RCC and that the other receptors may act as the compensatory receptor proteins to help ACE2. This suggests that increased expression of coronavirus receptors in ccRCC patients is correlated to an increased risk of case-related fatalities concerning healthy subjects. Finally, these receptors were associated with a high level of immune infiltration, inflammatory cytokines, various markers of exhausted T cells, and immunosuppressive microenvironment that highly correlated to coronavirus receptors in ccRCC patients. Therefore, these data suggested that coronavirus receptors may play an important role in modulating the immune infiltrate and cellular immunity and that the use of immune checkpoint inhibitor drugs could be the new treatment modality for ccRCC/COVID-19 patients [57]. Immune checkpoint inhibitors become an important therapeutic strategy for ccRCC with a beneficial effect on the prognosis of patients [92,93]. It has been observed that treatment with immune checkpoint inhibitors did not indicate an increased risk of adverse events in COVID-19 patients [77]. Correcting immune disorders seems to be a useful tool in ccRCC treatment, but immune checkpoint inhibitors are also important for treating COVID-19-positive patients.

During the pandemic, different medicinal plants/herbs and phytocompounds with immunomodulatory, anti-viral, and anti-inflammatory properties have been proposed in the treatment of COVID-19 [94]. A particular interest has been addressed to berberine in treating ccRCC/COVID-19 patients since it can inhibit SARS-CoV-2 infection and reproduction and reduce the inflammatory response in COVID-19 patients [95,96]. Recently some authors explored the berberine therapeutic mechanism in treating ccRCC/COVID-19 patients, and through gene ontology and protein-protein interaction (PPI) analysis, identified 26 target genes as potential berberine targets, involved in crucial biological processes in ccRCC/COVID-19 pathogenesis [97,98]. It has been reported that the severity of COVID-19 infection seemed to be strictly related to the over or downregulation of different genes involved in different roles of resisting viral infections [99], inflammation, and ROS production [100]. Thus, it has been postulated that these genes could be used as a prospective target for COVID-19 treatment.

In conclusion, this prognostic signature based on genes related to SARS-CoV-2 as reliable prognostic predictors for ccRCC patients is very promising and indicates a precise road of future research that is inseparable from massive sequencing studies.

## 6. Conclusions

**Figure 1:**
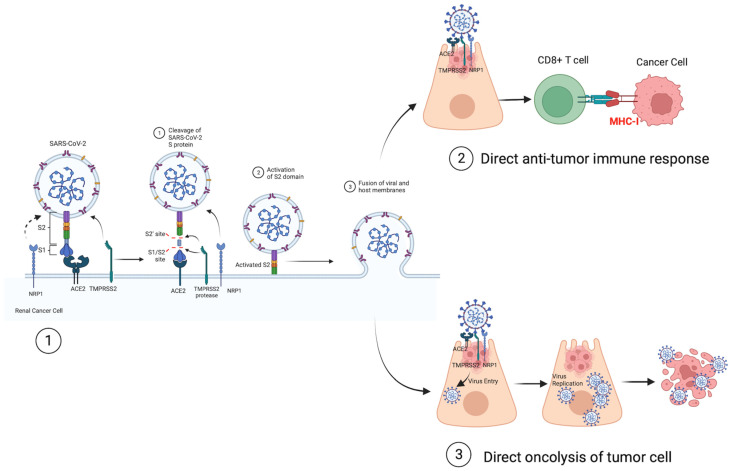
Potential mechanisms by which SARS-CoV-2 could promote renal cancer regression. (1) mediators of SARS-CoV-2 entry in tubular renal cells: glicoprotein S1 binds to the ACE2 receptor, glicoprotein S2 mediates the viral-cell membrane fusion by being exposed to TMPRSS2, NRP-1 interacs with furin-cleaved S1 glicoprotein, potentiating SARS-CoV-2 infectivity. (2) SARS-CoV-2 infects ACE2/TMPRSS2/NRP-1 expressing renal cancer cells promoting a direct antitumor immune response through cross-reactivity of viral-specific T cells with tumor antigens, (3) SARS-CoV-2 infects and replicates in renal cancer cell leading to deleterious alterations in cell function and, finally, killing tumor cell. SARS-CoV-2 spike (S) glycoproteins S1, S2; ACE2 (angiotensin-converting enzyme 2); TMPRSS2 (transmembrane protease serine 2); NPR1 (transmembrane receptor, neuropilin-1); TRC (T-cell receptor); MCH-I (Major Histocompatibility Complex Class I); CD8+ T Lymphocytes.

One of the most interesting aspects that have emerged from publications over the last three years concerning the link between SARS-CoV-2 infection and RCC is that renal cancer cells retain the key receptors allowing SARS-CoV-2 to entry in the host cell and that the virus could directly kill tumor cells or generate improved immunogenicity (Figure 1).

Though still preliminary, these experimental findings could open new scenarios in the treatment of renal cancer, as well as of other tumors, and, therefore, generate future study hypotheses. Furthermore, the identification of SARS-CoV-2-related genes and their pathways in RCC patients could represent a new prognostic tool to predict cancer progression and to develop new therapeutic strategies to counteract the complications of COVID-19-related and mortality risk.

Certainly, one of the limitations of this review, in addition to those arising from its non-systematic nature, is that the authors have mainly focused on the biomolecular aspects underlying the link between SARS-CoV-2 infection and renal cancer. On the other hand, the available clinical studies are limited and include cohorts of patients with small sample sizes, likely due to the low incidence of this tumor in the general population.

However, the evidence here described suggests continuing investigations on patients with histories of COVID-19 infections and kidney cancer in order to increase statistically significant correlations and identify new factors and different mechanisms that could further clarify the link between SARS-CoV-2 and RCC.

## References

[1] Shah W., Hillman T., Playford E.D., Hishmeh L. (2021). Managing the Long Term Effects of COVID-19: Summary of NICE, SIGN, and RCGP Rapid Guideline. BMJ.

[2] Nalbandian A., Sehgal K., Gupta A., Madhavan M.V., McGroder C., Stevens J.S., Cook J.R., Nordvig A.S., Shalev D., Sehrawat T.S. (2021). Post-Acute COVID-19 Syndrome. Nat. Med..

[3] Cummings M.J., Baldwin M.R., Abrams D., Jacobson S.D., Meyer B.J., Balough E.M., Aaron J.G., Claassen J., Rabbani L.E., Hastie J. (2020). Epidemiology, Clinical Course, and Outcomes of Critically Ill Adults with COVID-19 in New York City: A Prospective Cohort Study. Lancet.

[4] Chan L., Chaudhary K., Saha A., Chauhan K., Vaid A., Zhao S., Paranjpe I., Somani S., Richter F., Miotto R. (2021). AKI in Hospitalized Patients with COVID-19. J. Am. Soc. Nephrol..

[5] Hirsch J.S., Ng J.H., Ross D.W., Sharma P., Shah H.H., Barnett R.L., Hazzan A.D., Fishbane S., Jhaveri K.D., Northwell COVID-19 Research Consortium (2020). Acute Kidney Injury in Patients Hospitalized with COVID-19. Kidney Int..

[6] Kellum J.A., van Till J.W.O., Mulligan G. (2020). Targeting Acute Kidney Injury in COVID-19. Nephrol. Dial. Transpl..

[7] Raymond E., Thieblemont C., Alran S., Faivre S. (2020). Impact of the COVID-19 Outbreak on the Management of Patients with Cancer. Target Oncol..

[8] Akram N., Imran M., Noreen M., Ahmed F., Atif M., Fatima Z., Bilal Waqar A. (2017). Oncogenic Role of Tumor Viruses in Humans. Viral Immunol..

[9] White M.K., Pagano J.S., Khalili K. (2014). Viruses and Human Cancers: A Long Road of Discovery of Molecular Paradigms. Clin. Microbiol. Rev..

[10] Li Y.-S., Ren H.-C., Cao J.-H. (2022). Correlation of SARS-CoV-2 to Cancer: Carcinogenic or Anticancer? (Review). Int. J. Oncol..

[11] Kim J.-M., Kim H.M., Lee E.J., Jo H.J., Yoon Y., Lee N.-J., Son J., Lee Y.-J., Kim M.S., Lee Y.-P. (2020). Detection and Isolation of SARS-CoV-2 in Serum, Urine, and Stool Specimens of COVID-19 Patients from the Republic of Korea. Osong Public Health Res. Perspect..

[12] Elfiky A.A. (2020). Ribavirin, Remdesivir, Sofosbuvir, Galidesivir, and Tenofovir against SARS-CoV-2 RNA Dependent RNA Polymerase (RdRp): A Molecular Docking Study. Life Sci..

[13] Icard P., Lincet H., Wu Z., Coquerel A., Forgez P., Alifano M., Fournel L. (2021). The Key Role of Warburg Effect in SARS-CoV-2 Replication and Associated Inflammatory Response. Biochimie.

[14] Codo A.C., Davanzo G.G., de Brito Monteiro L., de Souza G.F., Muraro S.P., Virgilio-da-Silva J.V., Prodonoff J.S., Carregari V.C., de Biagi Junior C.A.O., Crunfli F. (2020). Elevated Glucose Levels Favor SARS-CoV-2 Infection and Monocyte Response through a HIF-1α/Glycolysis-Dependent Axis. Cell Metab..

[15] Dai M., Liu D., Liu M., Zhou F., Li G., Chen Z., Zhang Z., You H., Wu M., Zheng Q. (2020). Patients with Cancer Appear More Vulnerable to SARS-CoV-2: A Multicenter Study during the COVID-19 Outbreak. Cancer Discov..

[16] Yang K., Sheng Y., Huang C., Jin Y., Xiong N., Jiang K., Lu H., Liu J., Yang J., Dong Y. (2020). Clinical Characteristics, Outcomes, and Risk Factors for Mortality in Patients with Cancer and COVID-19 in Hubei, China: A Multicentre, Retrospective, Cohort Study. Lancet Oncol..

[17] Cheng Y., Luo R., Wang K., Zhang M., Wang Z., Dong L., Li J., Yao Y., Ge S., Xu G. (2020). Kidney Disease Is Associated with In-Hospital Death of Patients with COVID-19. Kidney Int..

[18] Peired A.J., Lazzeri E., Guzzi F., Anders H.-J., Romagnani P. (2021). From Kidney Injury to Kidney Cancer. Kidney Int..

[19] Jackson C.B., Farzan M., Chen B., Choe H. (2022). Mechanisms of SARS-CoV-2 Entry into Cells. Nat. Rev. Mol. Cell Biol..

[20] Khan S., Chen L., Yang C.-R., Raghuram V., Khundmiri S.J., Knepper M.A. (2020). Does SARS-CoV-2 Infect the Kidney?. J. Am. Soc. Nephrol..

[21] Rai V. (2023). COVID-19 and Kidney: The Importance of Follow-Up and Long-Term Screening. Life.

[22] Jonasch E., Gao J., Rathmell W.K. (2014). Renal Cell Carcinoma. BMJ.

[23] Chow W.-H., Dong L.M., Devesa S.S. (2010). Epidemiology and Risk Factors for Kidney Cancer. Nat. Rev. Urol..

[24] Banumathy G., Cairns P. (2010). Signaling Pathways in Renal Cell Carcinoma. Cancer Biol. Ther..

[25] Ganner A., Gehrke C., Klein M., Thegtmeier L., Matulenski T., Wingendorf L., Wang L., Pilz F., Greidl L., Meid L. (2021). VHL Suppresses RAPTOR and Inhibits mTORC1 Signaling in Clear Cell Renal Cell Carcinoma. Sci. Rep..

[26] Alonso-Gordoa T., García-Bermejo M.L., Grande E., Garrido P., Carrato A., Molina-Cerrillo J. (2019). Targeting Tyrosine Kinases in Renal Cell Carcinoma: “New Bullets against Old Guys”. Int. J. Mol. Sci..

[27] Dell’Atti L., Bianchi N., Aguiari G. (2022). New Therapeutic Interventions for Kidney Carcinoma: Looking to the Future. Cancers.

[28] Choong O.K., Jakobsson R., Bergdahl A.G., Brunet S., Kärmander A., Waldenström J., Arvidsson Y., Altiparmak G., Nilsson J.A., Karlsson J. (2023). SARS-CoV-2 Replicates and Displays Oncolytic Properties in Clear Cell and Papillary Renal Cell Carcinoma. PLoS ONE.

[29] Li Q., Oduro P.K., Guo R., Li R., Leng L., Kong X., Wang Q., Yang L. (2022). Oncolytic Viruses: Immunotherapy Drugs for Gastrointestinal Malignant Tumors. Front. Cell. Infect. Microbiol..

[30] Senapati S., Banerjee P., Bhagavatula S., Kushwaha P.P., Kumar S. (2021). Contributions of Human ACE2 and TMPRSS2 in Determining Host-Pathogen Interaction of COVID-19. J. Genet..

[31] Hossain M.G., Akter S., Uddin M.J. (2021). Emerging Role of Neuropilin-1 and Angiotensin-Converting Enzyme-2 in Renal Carcinoma-Associated COVID-19 Pathogenesis. Infect. Dis. Rep..

[32] Fedson D.S. (2023). Treating COVID-19: Targeting the Host Response, Not the Virus. Life.

[33] Gold S.A., Margulis V. (2023). Uncovering a Link between COVID-19 and Renal Cell Carcinoma. Nat. Rev. Urol..

[34] Hoffmann M. (2020). SARS-CoV-2 celle entry depends on ACE2 and TMPRSS2 and is blocked by a clinically proven protease inhibitor. Cell.

[35] Roca-Ho H., Riera M., Palau V., Pascual J., Soler M.J. (2017). Characterization of ACE and ACE2 Expression within Different Organs of the NOD Mouse. Int. J. Mol. Sci..

[36] Turner A.J., Nalivaeva N.N. (2022). Angiotensin-Converting Enzyme 2 (ACE2): Two Decades of Revelations and Re-Evaluation. Peptides.

[37] Samavati L., Uhal B.D. (2020). ACE2, Much More Than Just a Receptor for SARS-COV-2. Front. Cell. Infect. Microbiol..

[38] Martyniak A., Tomasik P.J. (2022). A New Perspective on the Renin-Angiotensin System. Diagnostics.

[39] Sang E.R., Tian Y., Miller L.C., Sang Y. (2021). Epigenetic Evolution of ACE2 and IL-6 Genes: Non-Canonical Interferon-Stimulated Genes Correlate to COVID-19 Susceptibility in Vertebrates. Genes.

[40] Saleh A., Sultan A., Elashry M.A., Farag A., Mortada M.I., Ghannam M.A., Saed A.M., Ghoneem E. (2022). Association of TNF-α G-308 a Promoter Polymorphism with the Course and Outcome of COVID-19 Patients. Immunol. Investig..

[41] Heinzelman P., Romero P.A. (2023). Directed Evolution of Angiotensin-Converting Enzyme 2 Peptidase Activity Profiles for Therapeutic Applications. Protein Sci..

[42] Mizuiri S., Ohashi Y. (2015). ACE and ACE2 in Kidney Disease. World J. Nephrol..

[43] Fan C., Lu W., Li K., Ding Y., Wang J. (2020). ACE2 Expression in Kidney and Testis May Cause Kidney and Testis Infection in COVID-19 Patients. Front. Med..

[44] Muglia V.F., Prando A. (2015). Renal Cell Carcinoma: Histological Classification and Correlation with Imaging Findings. Radiol. Bras..

[45] Niu X., Zhu Z., Shao E., Bao J. (2021). ACE2 Is a Prognostic Biomarker and Associated with Immune Infiltration in Kidney Renal Clear Cell Carcinoma: Implication for COVID-19. J. Oncol..

[46] Zhang S., Zhang E., Long J., Hu Z., Peng J., Liu L., Tang F., Li L., Ouyang Y., Zeng Z. (2019). Immune Infiltration in Renal Cell Carcinoma. Cancer Sci..

[47] Khanna P., Soh H.J., Chen C.-H., Saxena R., Amin S., Naughton M., Joslin P.N., Moore A., Bakouny Z., O’Callaghan C. (2021). ACE2 Abrogates Tumor Resistance to VEGFR Inhibitors Suggesting Angiotensin-(1-7) as a Therapy for Clear Cell Renal Cell Carcinoma. Sci. Transl. Med..

[48] Yang X., Lin C., Liu J., Zhang Y., Deng T., Wei M., Pan S., Lu L., Li X., Tian G. (2023). Identification of the Regulatory Mechanism of ACE2 in COVID-19-Induced Kidney Damage with Systems Genetics Approach. J. Mol. Med..

[49] Meiners J., Jansen K., Gorbokon N., Büscheck F., Luebke A.M., Kluth M., Hube-Magg C., Höflmayer D., Weidemann S., Fraune C. (2021). Angiotensin-Converting Enzyme 2 Protein Is Overexpressed in a Wide Range of Human Tumour Types: A Systematic Tissue Microarray Study on >15,000 Tumours. Biomedicines.

[50] Tang Q., Wang Y., Ou L., Li J., Zheng K., Zhan H., Gu J., Zhou G., Xie S., Zhang J. (2021). Downregulation of ACE2 Expression by SARS-CoV-2 Worsens the Prognosis of KIRC and KIRP Patients via Metabolism and Immunoregulation. Int. J. Biol. Sci..

[51] Mia M.S., Hossain D., Woodbury E., Kelleher S., Palamuttam R.J., Rao R., Steen P., Jarajapu Y.P., Mathew S. (2023). Integrin Β1 Is a Key Determinant of the Expression of Angiotensin-Converting Enzyme 2 (ACE2) in the Kidney Epithelial Cells. Eur. J. Cell. Biol..

[52] Ottaiano A., Scala S., D’Alterio C., Trotta A., Bello A., Rea G., Picone C., Santorsola M., Petrillo A., Nasti G. (2021). Unexpected Tumor Reduction in Metastatic Colorectal Cancer Patients during SARS-Cov-2 Infection. Ther. Adv. Med. Oncol..

[53] Barkhordar M., Rostami F.T., Yaghmaie M., Abbaszadeh M., Chahardouli B., Mousavi S.A. (2022). Spontaneous Complete Remission of Acute Myeloid Leukemia in the Absence of Disease-Modifying Therapy Following Severe Pulmonary Involvement by Coronavirus Infectious Disease-19. Case Rep. Hematol..

[54] Bounassar-Filho J.P., Boeckler-Troncoso L., Cajigas-Gonzalez J., Zavala-Cerna M.G. (2023). SARS-CoV-2 as an Oncolytic Virus Following Reactivation of the Immune System: A Review. Int. J. Mol. Sci..

[55] Liu L., Qin J.-F., Zuo M.-Z., Zhou Q. (2022). Multi-Omics of the Expression and Clinical Outcomes of TMPRSS2 in Human Various Cancers: A Potential Therapeutic Target for COVID-19. J. Cell. Mol. Med..

[56] Sacconi A., Donzelli S., Pulito C., Ferrero S., Spinella F., Morrone A., Rigoni M., Pimpinelli F., Ensoli F., Sanguineti G. (2020). TMPRSS2, a SARS-CoV-2 Internalization Protease Is Downregulated in Head and Neck Cancer Patients. J. Exp. Clin. Cancer Res..

[57] Tripathi S.C., Deshmukh V., Creighton C.J., Patil A. (2020). Renal Carcinoma Is Associated with Increased Risk of Coronavirus Infections. Front. Mol. Biosci..

[58] Mihalopoulos M., Dogra N., Mohamed N., Badani K., Kyprianou N. (2020). COVID-19 and Kidney Disease: Molecular Determinants and Clinical Implications in Renal Cancer. Eur. Urol. Focus.

[59] Zhou Y., Liu Y., Gupta S., Paramo M.I., Hou Y., Mao C., Luo Y., Judd J., Wierbowski S., Bertolotti M. (2023). A Comprehensive SARS-CoV-2-Human Protein-Protein Interactome Reveals COVID-19 Pathobiology and Potential Host Therapeutic Targets. Nat. Biotechnol..

[60] Roos F.C., Roberts A.M., Hwang I.I.L., Moriyama E.H., Evans A.J., Sybingco S., Watson I.R., Carneiro L.A.M., Gedye C., Girardin S.E. (2010). Oncolytic Targeting of Renal Cell Carcinoma via Encephalomyocarditis Virus. EMBO Mol. Med..

[61] Lawson K.A., Morris D.G. (2012). Oncolytic Virotherapy for Renal Cell Carcinoma: A Novel Treatment Paradigm?. Expert Opin. Biol. Ther..

[62] Breitbach C.J., Bell J.C., Hwang T.-H., Kirn D.H., Burke J. (2015). The Emerging Therapeutic Potential of the Oncolytic Immunotherapeutic Pexa-Vec (JX-594). Oncolytic Virother..

[63] Chulpanova D.S., Kitaeva K.V., Green A.R., Rizvanov A.A., Solovyeva V.V. (2020). Molecular Aspects and Future Perspectives of Cytokine-Based Anti-Cancer Immunotherapy. Front. Cell. Dev. Biol..

[64] Fang L., Tian W., Zhang C., Wang X., Li W., Zhang Q., Zhang Y., Zheng J. (2023). Oncolytic Adenovirus-Mediated Expression of CCL5 and IL12 Facilitates CA9-Targeting CAR-T Therapy against Renal Cell Carcinoma. Pharmacol. Res..

[65] Taguchi S., Fukuhara H., Homma Y., Todo T. (2017). Current Status of Clinical Trials Assessing Oncolytic Virus Therapy for Urological Cancers. Int. J. Urol..

[66] Castelo-Branco L., Tsourti Z., Gennatas S., Rogado J., Sekacheva M., Viñal D., Lee R., Croitoru A., Vitorino M., Khallaf S. (2022). COVID-19 in Patients with Cancer: First Report of the ESMO International, Registry-Based, Cohort Study (ESMO-CoCARE). ESMO Open.

[67] Brown M.C., Holl E.K., Boczkowski D., Dobrikova E., Mosaheb M., Chandramohan V., Bigner D.D., Gromeier M., Nair S.K. (2017). Cancer Immunotherapy with Recombinant Poliovirus Induces IFN-Dominant Activation of Dendritic Cells and Tumor Antigen-Specific CTLs. Sci. Transl. Med..

[68] ESMO Cancer Patient Management during the COVID-19 Pandemic. https://www.esmo.org/guidelines/cancer-patient-management-during-the-covid-19-pandemic.

[69] Bakouny Z., Paciotti M., Schmidt A.L., Lipsitz S.R., Choueiri T.K., Trinh Q.-D. (2021). Cancer Screening Tests and Cancer Diagnoses during the COVID-19 Pandemic. JAMA Oncol..

[70] Patt D., Gordan L., Diaz M., Okon T., Grady L., Harmison M., Markward N., Sullivan M., Peng J., Zhou A. (2020). Impact of COVID-19 on Cancer Care: How the Pandemic Is Delaying Cancer Diagnosis and Treatment for American Seniors. JCO Clin. Cancer Inform..

[71] Roy P., van Peer S.E., Dandis R., Duncan C., de Aguirre-Neto J.C., Verschuur A., de Camargo B., Karim-Kos H.E., Boschetti L., Spreafico F. (2023). Impact of the COVID-19 Pandemic on Paediatric Renal Tumour Presentation and Management, a SIOP Renal Tumour Study Group Study. Cancer Med..

[72] Velavan T.P., Pallerla S.R., Rüter J., Augustin Y., Kremsner P.G., Krishna S., Meyer C.G. (2021). Host Genetic Factors Determining COVID-19 Susceptibility and Severity. eBioMedicine.

[73] Ellinghaus D., Degenhardt F., Bujanda L., Buti M., Albillos A., Invernizzi P., Fernández J., Prati D., Baselli G., Severe COVID-19 GWAS Group (2020). Genomewide Association Study of Severe COVID-19 with Respiratory Failure. N. Engl. J. Med..

[74] Lennon H., Sperrin M., Badrick E., Renehan A.G. (2016). The Obesity Paradox in Cancer: A Review. Curr. Oncol. Rep..

[75] Robilotti E.V., Babady N.E., Mead P.A., Rolling T., Perez-Johnston R., Bernardes M., Bogler Y., Caldararo M., Figueroa C.J., Glickman M.S. (2020). Determinants of COVID-19 Disease Severity in Patients with Cancer. Nat. Med..

[76] Rogiers A., Pires da Silva I., Tentori C., Tondini C.A., Grimes J.M., Trager M.H., Nahm S., Zubiri L., Manos M., Bowling P. (2021). Clinical Impact of COVID-19 on Patients with Cancer Treated with Immune Checkpoint Inhibition. J. Immunother. Cancer.

[77] Mandala M., Lorigan P., De Luca M., Bianchetti A., Merelli B., Bettini A.C., Bonomi L., Nahm S., Vitale M.G., Negrini G. (2021). SARS-CoV-2 Infection and Adverse Events in Patients with Cancer Receiving Immune Checkpoint Inhibitors: An Observational Prospective Study. J. Immunother. Cancer.

[78] Luo J., Rizvi H., Egger J.V., Preeshagul I.R., Wolchok J.D., Hellmann M.D. (2020). Impact of PD-1 Blockade on Severity of COVID-19 in Patients with Lung Cancers. Cancer Discov..

[79] Hasanov E., Gao J., Tannir N.M. (2020). The Immunotherapy Revolution in Kidney Cancer Treatment: Scientific Rationale and First-Generation Results. Cancer J..

[80] Xu W., Atkins M.B., McDermott D.F. (2020). Checkpoint Inhibitor Immunotherapy in Kidney Cancer. Nat. Rev. Urol..

[81] Perazella M.A., Shirali A.C. (2018). Nephrotoxicity of Cancer Immunotherapies: Past, Present and Future. J. Am. Soc. Nephrol..

[82] Schoenfeld A.J., Hellmann M.D. (2020). Acquired Resistance to Immune Checkpoint Inhibitors. Cancer Cell.

[83] De Giglio A., Di Federico A., Nuvola G., Deiana C., Gelsomino F. (2021). The Landscape of Immunotherapy in Advanced NSCLC: Driving Beyond PD-1/PD-L1 Inhibitors (CTLA-4, LAG3, IDO, OX40, TIGIT, Vaccines). Curr. Oncol. Rep..

[84] Tsimafeyeu I., Alekseeva G., Berkut M., Nosov A., Myslevtsev I., Andrianov A., Semenov A., Borisov P., Zukov R., Goutnik V. (2021). COVID-19 in Patients with Renal Cell Carcinoma in the Russian Federation. Clin. Genitourin. Cancer.

[85] Szabados B., Abu-Ghanem Y., Grant M., Choy J., Bex A., Powles T. (2020). Clinical Characteristics and Outcome for Four SARS-CoV-2-Infected Cancer Patients Treated with Immune Checkpoint Inhibitors. Eur. Urol..

[86] García-Donas J., de Velasco G., Madurga R., Chamorro J., Rosero D., Etxaniz O., Pérez-Gracia J.L., Pinto Á., Cacho D., Barba M. (2023). Case-Control Study Assessing the Impact of COVID19 in Advanced Kidney Cancer Patients Treated with Antiangiogenics or Immunotherapy: The COVID-REN Study. Clin. Transl. Oncol..

[87] Huang Y., Chen S., Xiao L., Qin W., Li L., Wang Y., Ma L., Yuan X. (2021). A Novel Prognostic Signature for Survival Prediction and Immune Implication Based on SARS-CoV-2-Related Genes in Kidney Renal Clear Cell Carcinoma. Front. Bioeng. Biotechnol..

[88] Gudas L.J., Fu L., Minton D.R., Mongan N.P., Nanus D.M. (2014). The Role of HIF1α in Renal Cell Carcinoma Tumorigenesis. J. Mol. Med..

[89] Khalil B.A., Elemam N.M., Maghazachi A.A. (2021). Chemokines and Chemokine Receptors during COVID-19 Infection. Comput. Struct. Biotechnol. J..

[90] Tian M., Liu W., Li X., Zhao P., Shereen M.A., Zhu C., Huang S., Liu S., Yu X., Yue M. (2021). HIF-1α Promotes SARS-CoV-2 Infection and Aggravates Inflammatory Responses to COVID-19. Signal Transduct. Target. Ther..

[91] Abdulla Alwaili M. (2023). Transcriptomic Analysis in Renal Cell Carcinoma and COVID-19 Patients. Cell. Mol. Biol..

[92] Thouvenin J., Alhalabi O., Carlo M., Carril-Ajuria L., Hirsch L., Martinez-Chanza N., Négrier S., Campedel L., Martini D., Borchiellini D. (2022). Efficacy of Cabozantinib in Metastatic MiT Family Translocation Renal Cell Carcinomas. Oncologist.

[93] Chen Y.-W., Tucker M.D., Brown L.C., Yasin H.A., Ancell K.K., Armstrong A.J., Beckermann K.E., Davis N.B., Harrison M.R., Kaiser E.G. (2022). The Association between a Decrease in On-Treatment Neutrophil-to-Eosinophil Ratio (NER) at Week 6 after Ipilimumab Plus Nivolumab Initiation and Improved Clinical Outcomes in Metastatic Renal Cell Carcinoma. Cancers.

[94] Akter R., Rahman M.R., Ahmed Z.S., Afrose A. (2023). Plausibility of Natural Immunomodulators in the Treatment of COVID-19-A Comprehensive Analysis and Future Recommendations. Heliyon.

[95] Lopes T.Z., de Moraes F.R., Tedesco A.C., Arni R.K., Rahal P., Calmon M.F. (2020). Berberine Associated Photodynamic Therapy Promotes Autophagy and Apoptosis via ROS Generation in Renal Carcinoma Cells. Biomed. Pharmacother..

[96] Zhang B.Y., Chen M., Chen X.C., Cao K., You Y., Qian Y.J., Yu W.K. (2021). Berberine Reduces Circulating Inflammatory Mediators in Patients with Severe COVID-19. Br. J. Surg..

[97] Xu Z., Chen S., Liu R., Chen H., Xu B., Xu W., Chen M. (2022). Circular RNA circPOLR2A Promotes Clear Cell Renal Cell Carcinoma Progression by Facilitating the UBE3C-Induced Ubiquitination of PEBP1 and, Thereby, Activating the ERK Signaling Pathway. Mol. Cancer.

[98] Appelberg S., Gupta S., Svensson Akusjärvi S., Ambikan A.T., Mikaeloff F., Saccon E., Végvári Á., Benfeitas R., Sperk M., Ståhlberg M. (2020). Dysregulation in Akt/mTOR/HIF-1 Signaling Identified by Proteo-Transcriptomics of SARS-CoV-2 Infected Cells. Emerg. Microbes Infect..

[99] Gutman H., Aftalion M., Melamed S., Politi B., Nevo R., Havusha-Laufer S., Achdout H., Gur D., Israely T., Dachir S. (2022). Matrix Metalloproteinases Expression Is Associated with SARS-CoV-2-Induced Lung Pathology and Extracellular-Matrix Remodeling in K18-hACE2 Mice. Viruses.

[100] Chung H.-L., Wangler M.F., Marcogliese P.C., Jo J., Ravenscroft T.A., Zuo Z., Duraine L., Sadeghzadeh S., Li-Kroeger D., Schmidt R.E. (2020). Loss- or Gain-of-Function Mutations in ACOX1 Cause Axonal Loss via Different Mechanisms. Neuron.

